# Using single-molecule fluorescence *in situ* hybridization and immunohistochemistry to count RNA molecules in single cells in zebrafish embryos

**DOI:** 10.1016/j.xpro.2022.102020

**Published:** 2023-01-12

**Authors:** Kemal Keseroglu, Oriana Q.H. Zinani, Ertuğrul M. Özbudak

**Affiliations:** 1 Division of Developmental Biology, Cincinnati Children’s Hospital Medical Center, Cincinnati, OH 45229, USA; 2 Molecular and Developmental Biology Graduate Program, University of Cincinnati, College of Medicine, Cincinnati, OH 45229, USA; 3 Department of Pediatrics, University of Cincinnati College of Medicine, Cincinnati, OH 45229, USA

**Keywords:** Single-molecule Assays, Cell Biology, Developmental biology, Microscopy, Model Organisms, Molecular Biology, In Situ Hybridization, Molecular/Chemical Probes

## Abstract

Taming gene expression variability is critical for robust pattern formation during embryonic development. Here, we describe an optimized protocol for single-molecule fluorescence *in situ* hybridization and immunohistochemistry in zebrafish embryos. We detail how to count segmentation clock RNAs and calculate their variability among neighboring cells. This approach is easily adaptable to count RNA numbers of any gene and calculate transcriptional variability among neighboring cells in diverse biological settings.

For complete details on the use and execution of this protocol, please refer to Keskin et al. (2018),^1^ Zinani et al. (2021),^2^ and Zinani et al. (2022).^3^

## Highlights

• Optimized protocol for smFISH combined with immunohistochemistry in zebrafish embryos
• Sample preparation for confocal imaging of single transcripts
• Quantifying RNA molecules in single cells in a given tissue
• Quantifying transcriptional variability among neighboring cells

## Abstract (editor highlights)

Publisher’s note: Undertaking any experimental protocol requires adherence to local institutional guidelines for laboratory safety and ethics.

## Graphical abstract

**Figure:**
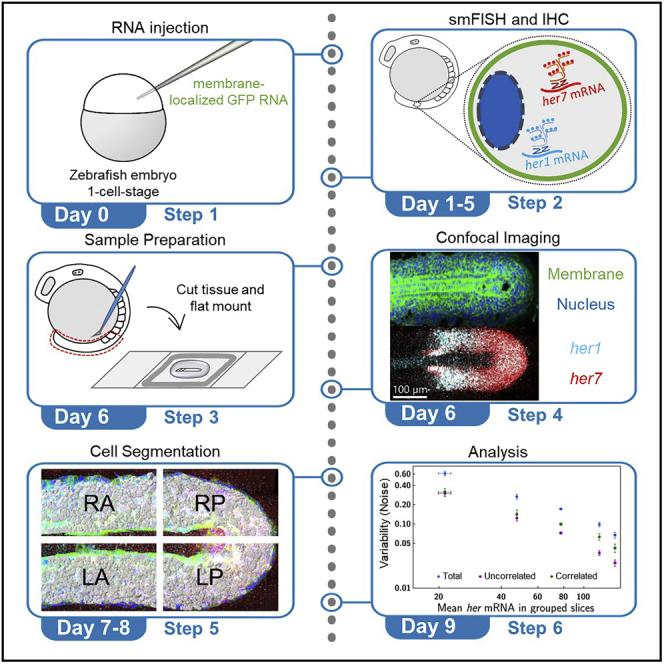

## Before you begin

### Institutional permissions

In this protocol, we used AB wild-type line, *her1*^*ci301*^;*her7*^*hu2526*^ and *Df(Chr05:her1,her7,ndrg3a)b567* mutant lines.^2^^,^^4^ The zebrafish experiments were performed under the ethical guideline of Cincinnati Children’s Hospital Medical Center. The animal protocol was reviewed and approved by Cincinnati Children’s Hospital Medical Center Animal Care and Use Committees (Protocol # 2020-0031). All zebrafish experiments must comply with protocols approved by a local animal ethics committee.

### Order single-molecule fluorescence *in situ* hybridization (smFISH) probes

To target specific mRNAs, order probes from ACDBio. Make sure they are assigned to different channels and compatible with other fluorophores that you are going to use for labeling. In this protocol, *her1* and *her7* mRNAs are labeled together with membrane-GFP by immunohistochemistry (IHC) and nucleus by Hoechst staining. Thus, “*Dr-her1-LE2*-C3” and “*Dr-her7*-C1” RNAscope® probes are ordered, respectively (Table 1).

**Table 1:** Amp 4 Alt B fluorescent label combination for the specific experimental design

Target (channel ID)	Staining/probe	Color
*her1* (C3)	Atto 647 (*Dr-her1-LE2*-C3)	Far Red
*her7* (C1)	Atto 550 (*Dr-her7*)	Red
Nucleus	Hoechst	Blue
Membrane	GFP	Green

### Membrane-GFP RNA *in vitro* transcription

**Timing: 5 h**

1. Linearize pCS2+EGFP-CAAX plasmid by *NotI.*
2. Purify DNA and elute in RNase free water.
3. Prepare membrane-localized GFP RNA using SP6 mMessage mMachine.
4. Purify RNA with lithium chloride precipitation and resuspend in RNase free water.
5. Make sure the concentration of RNA product is equal or higher than 400 ng/μL because it is going to be injected as 400 ng/μL.

***Alternatives:*** You can use any plasmid that contains the sequence of membrane-localized GFP. If so, use proper kit suitable for promoter (i.e., SP6, T7 or T3).

### Fish set up

**Timing: 30 min**

6. Set up breeding pairs in separate tanks one night before the injection day.
  a. Homozygous *her1*^*ci301*^*;her7*^*hu2526*^ fish are placed into a breeding tank.
    ***Alternatives:*** You can use any transgenic or mutant fish line.
  b. Male and female fish are separated by a divider, which enables to control breeding time.

### Membrane-GFP injection

**Timing: 1 h**

7. Prepare 1 μL (400 ng/μL) of membrane-localized GFP RNA. Keep it in ice.
8. Load mRNA into injection needle.
9. Set the volume of injection to 1 nL.
10. Transfer fish into a new breeding tank with fresh water and remove the divider.
11. Collect embryos after 10 min.
12. Inject 400 pg of membrane-GFP mRNA into one-cell stage embryos.
13. Incubate embryos at desired temperature for 12–20 h.

***Note:*** In order to fix embryos at 12–14 somite stage in the morning the day after, incubate embryos at 28°C until the shield stage, then transfer them to 23°C and incubate them for 12–16 h.

***Optional:*** Based on the experimental purpose, one can incubate the embryos between 21.5°C and 32°C until they reach the desired developmental stage.

## Key resources table

**Table:** 

REAGENT or RESOURCE	SOURCE	IDENTIFIER
**Antibodies**		

Chicken IgY, anti-GFP, unconjugated, Primary Antibody (2 mg/mL) (200×)	Fisher Scientific	Cat#A10262, RRID: AB_2534023
Alexa Fluor 488 Goat anti-Chicken IgG (H+L) Secondary Antibody (2 mg/mL) (200×)	Fisher Scientific	Cat#A-11039; RRID: AB_142924

**Bacterial and virus strains**		

pCS2+EGFP-CAAX	Keskin et al.^1^	N/A

**Chemicals, peptides, and recombinant proteins**		

FastDigest NotI	Fisher Scientific	Cat#FD0596
SP6 mMessage mMachine	Life Technologies	Cat#AM1340
Sodium chloride (NaCl)	Fisher Scientific	Cat#BP358-212
Potassium chloride (KCl)	Sigma-Aldrich	Cat#793590
Sodium phosphate, dibasic, heptahydrate (Na_2_HPO_4_)	Fisher Scientific	Cat#BP332-500
Potassium dihydrogen phosphate (KH_2_PO_4_)	Fisher Scientific	Cat#BP363-500
Sodium hydroxide (NaOH)	Fisher Scientific	Cat#BP359-500
DEPC-treated nuclease-free water	Growcells	Cat#UPW-010L
Paraformaldehyde (PFA)	Sigma-Aldrich	Cat#158127-500G
Sodium citrate dihydrate (Na_3_C_6_H_5_O_7_ · 2H_2_O)	Fisher Scientific	Cat#BP327-500
Ribonucleic acid from torula yeast (tRNA)	Sigma-Aldrich	Cat#R6625-100G
Urea	Sigma-Aldrich	Cat#U5378-100G
Tween 20	Sigma-Aldrich	Cat#P1379-500ML
Triton X-100	Sigma-Aldrich	Cat#X100-100ML
Citric acid	Fisher Scientific	Cat#A940-500
Heparin salt	Sigma-Aldrich	Cat#H4784-1G
Bovine serum albumin (BSA)	Sigma-Aldrich	Cat#A3294-100G
Goat serum, New Zealand origin (GS)	Fisher Scientific	Cat#16210064
Methanol	Sigma-Aldrich	Cat#179337-4L
RNAscope Fluorescent Multiplex Detection Reagents	Advanced Cell Diagnostics	Cat#320851
RNAscope Protease III Reagents	Advanced Cell Diagnostics	Cat#322340
RNAscope Probe - *Dr-her1-LE2-*C3	Advanced Cell Diagnostics	Cat#433201-C3
RNAscope Probe - *Dr-her7*	Advanced Cell Diagnostics	Cat#428611
Hoechst trihydrochloride, trihydrate (10 mg/mL)	Invitrogen	Cat#H3570
ProLong Gold antifade reagent	Life Technologies	Cat#P36934

**Deposited data**		

Image Processing Pipeline	This paper	https://github.com/ozbudak/STARMethods_2022
Raw and analyzed data related to *her1^ci301^*;*her7^hu2526^* and *her1^b567/ci301^*;*her7^b567/hu2526^* mutant embryos	Zinani et al.^3^	https://www.ebi.ac.uk/biostudies/studies/S-BSST847
Raw and analyzed data related to wild-type embryos	Zinani et al.^2^	https://www.ebi.ac.uk/biostudies/studies/S-BSST434

**Experimental models: Organisms/strains**		

Zebrafish: *her1*^*ci301*^;*her7*^*hu2526*^	Zinani et al.^2^	ZFIN ID: ZDB-ALT-211025-4
Zebrafish: *Df(Chr05:her1,her7,ndrg3a)b567*	Henry et al.^4^	ZFIN ID: ZDB-ALT-030512-2

**Software and algorithms**		

Imaris 9.8	Bitplane	http://www.bitplane.com/imaris/imaris; RRID: SCR_007370
Python Programming Language, version 3.8	Python Software Foundation	http://www.python.org/; RRID: SCR_008394
Matlab_R2020b	Mathworks	http://www.mathworks.com/products/matlab/; RRID: SCR_001622

**Other**		

Nikon A1R GaAsP inverted confocal microscope 100 × 1.49 NA Apo TIRF DIC- Oil objective	Nikon	N/A
500 mL vacuum filter/storage bottle system	Corning	Cat#430758
1.5 mL micro centrifuge tube	Fisher Scientific	Cat#14-666-319
2 mL micro centrifuge tube	Fisher Scientific	Cat#14-666-315
Glass Pasteur pipets (cotton-plugged)	Fisher Scientific	Cat#13-678-8B
Pipette pump	Fisher Scientific	Cat#13-683C
Petri dish (100 mm × 15 mm)	Fisher Scientific	Cat#FB087579B
Oven	Fisher Scientific	Cat#HBSNSRS110
Rotary shaker	N/A	N/A

## Materials and equipment

**Table:** 10× Phosphate buffered saline (PBS)

Reagent	Final concentration	Amount
NaCl	1.37 M	56.04 g
KCl	27 mM	1.41 g
Na_2_HPO_4_	100 mM	9.94 g
KH_2_PO_4_	18 mM	1.72 g
DEPC H_2_O	N/A	Up to 700 mL
**Total**	**N/A**	**700 mL**

10× PBS may be stored at 20°C–25°C for several months.

***Note:*** Adjust pH to 7.4 then filter in a sterile storage bottle by vacuum filtering with 0.22 μm pore.

**Table:** 4% Paraformaldehyde (4% PFA)

Reagent	Final concentration	Amount
10× PBS	1× PBS	50 mL
Paraformaldehyde	4% (weight/volume)	20 g
DEPC H_2_O	N/A	Up to 500 mL
**Total**	**N/A**	**500 mL**

4% PFA may be stored at −20°C for several months, and 4°C for no more than 1 week.

For 500 mL 4% PFA, add 50 mL 10× PBS and 400 mL DEPC H_2_O to a flask on hot plate stirrer in a ventilated hood. Add a stir bar and stir at around 60°C and 700 rpm in the hood. Add 20 g paraformaldehyde powder to the heated PBS solution. Continue to stir and slowly add 1 N NaOH until the solution clears. Once the paraformaldehyde is completely dissolved, cool the solution down, and adjust the volume to 500 mL with DEPC H_2_O, and pH to 7.0. Then filter in a sterile storage bottle by vacuum filtering with 0.22 μm pore. Divide in aliquots of 15 mL tubes, and store at −20°C. Avoid multiple freeze and thaw cycles.

**CRITICAL:** Paraformaldehyde is flammable, toxic and potentially carcinogenic, and causes eye, skin, and respiratory tract irritation. Should be handled in a fume hood with eye and respiratory protection.

**Table:** PBST-0.1%

Reagent	Final concentration	Amount
10× PBS	1× PBS	5 mL
DEPC H_2_O	N/A	Up to 50 mL
Tween 20	0.1% (volume/volume)	50 μL
**Total**	**N/A**	**50 mL**

Prepare fresh PBST0.1% on the day of experiment and leave it at 20°C–25°C.

**Table:** PBST-0.01%

Reagent	Final concentration	Amount
10× PBS	1× PBS	5 mL
DEPC H_2_O	N/A	Up to 50 mL
Tween 20	0.01% (volume/volume)	5 μL
**Total**	**N/A**	**50 mL**

Prepare fresh PBST0.01% on the day of experiment and leave it at 20°C–25°C.

**Table:** PBSTX

Reagent	Final concentration	Amount
10× PBS	1× PBS	5 mL
DEPC H_2_O	N/A	Up to 50 mL
Triton X-100	1% (volume/volume)	500 μL
**Total**	**N/A**	**50 mL**

Fresh PBSTX needs to be prepared one day before the experiment and used on the following days. PBSTX may be stored at 20°C–25°C for 1 week.

***Note:*** To avoid bubbles, prepare PBSTX with (5 mL 10×PBS + 500 μL Triton X-100) ∼30 mL DEPC H_2_O one day before the experiment and leave it at 20°C–25°C. Top it off to 50 mL with DEPC H_2_O and use it on the day of experiment, Day 4.

**Table:** Permeabilization buffer

Reagent	Final concentration	Amount
10× PBS	1× PBS	100 μL
DEPC H_2_O	N/A	885 μL
Triton X-100	1.5% (volume/volume)	15 μL
**Total**	**N/A**	**1 mL**

Prepare fresh permeabilization buffer on the day of experiment and leave it at 20°C–25°C.

***Optional:*** You can also prepare permeabilization buffer using PBSTX: 995 μL PBSTX + 5 μL Triton X-100.

**Table:** PBSTXBG

Reagent	Final concentration	Amount
10× PBS	1× PBS	1 mL
Bovine Serum Albumin (BSA)	2% (weight/volume)	0.2 g
Goat Serum (GS)	1% (volume/volume)	100 μL
DEPC H_2_O	N/A	Up to 10 mL
Triton X-100	1% (volume/volume)	100 μL
**Total**	**N/A**	**10 mL**

Prepare fresh PBSTXBG on the day of experiment and keep it on ice. Store at 4°C for 1 day.

***Optional:*** You can also prepare PBSTXBG using PBSTX: 0.2 g BSA + 10 mL PBSTX + 100 μL GS.

**Table:** 20× Saline-sodium citrate buffer (SSC)

Reagent	Final concentration	Amount
NaCl	3 M	87.66 g
Sodium Citrate Dihydrate	0.3 M	44.12 g
DEPC H_2_O	N/A	Up to 500 mL
**Total**	**N/A**	**500 mL**

20× SSC may be stored at 20°C–25°C for several months.

***Note:*** Adjust pH to 7.0 then filter in a sterile storage bottle by vacuum filtering with 0.22 μm pore.

**Table:** SSCT

Reagent	Final concentration	Amount
20× SSC	0.2× SSC	500 μL
DEPC H_2_O	N/A	Up to 50 mL
Tween 20	0.01% (volume/volume)	5 μL
**Total**	**N/A**	**50 mL**

Prepare fresh SSCT on the day of experiment and leave it at 20°C–25°C.

**Table:** Prehybridization buffer

Reagent	Final concentration	Amount
20× SSC	5× SSC	2.5 mL
tRNA	0.05 mg/μL	5.6 mg
Urea	0.05 mg/μL	2.4 g
Tween 20	0.01% (volume/volume)	10 μL
Citric Acid (1 M pH 6.0)	9.2 mM	92 μL
Heparin Salt (5 mg/μL)	0.05 mg/μL	100 μL
DEPC H_2_O	N/A	Up to 10 mL
**Total**	**N/A**	**10 mL**

Prepare fresh prehybridization buffer on the day of experiment and keep it on ice.

## Step-by-step method details

### Day 1: Fixation

**Timing: 3 h (for step 1)**

This section describes the steps for fixation and dehydration of embryos on the first day of experiment.

1. Wait until the embryos reach 12–14 somite stage.

***Note:*** Before start fixation, thaw one aliquot of 4% PFA and make sure it is at 20°C–25°C. Embryos would be around 10–11 somite stage at 9 am if they were incubated at 28°C until the shield stage and then incubated at 23°C for 12–16 h. Check GFP signal and morphology of embryos under fluorescence microscope. Remove embryos with abnormal morphology or weak GFP signal. Select 15 embryos for each experimental condition. Always keep embryos in dark; either cover tubes with aluminum foil or keep them in a dark box until the end of staining.

***Note:*** Embryos would be ready to be fixed after removal of ones with abnormal morphology or weak GFP signal. But depending on the injection room temperature (21°C–28°C), embryos might be earlier than 10 somite stage at 9 am, and it might take 1–2 h to reach 12 somite stage.

2. Transfer embryos into 2 mL tube. Remove excess water.
3. Rinse with 1 mL 4% PFA (add and then remove 1 mL 4% PFA).
4. Add 2 mL 4% PFA. Cover the tube to keep embryos in dark and shake embryos for 1 h at 20°C–25°C.

**Figure 1:**
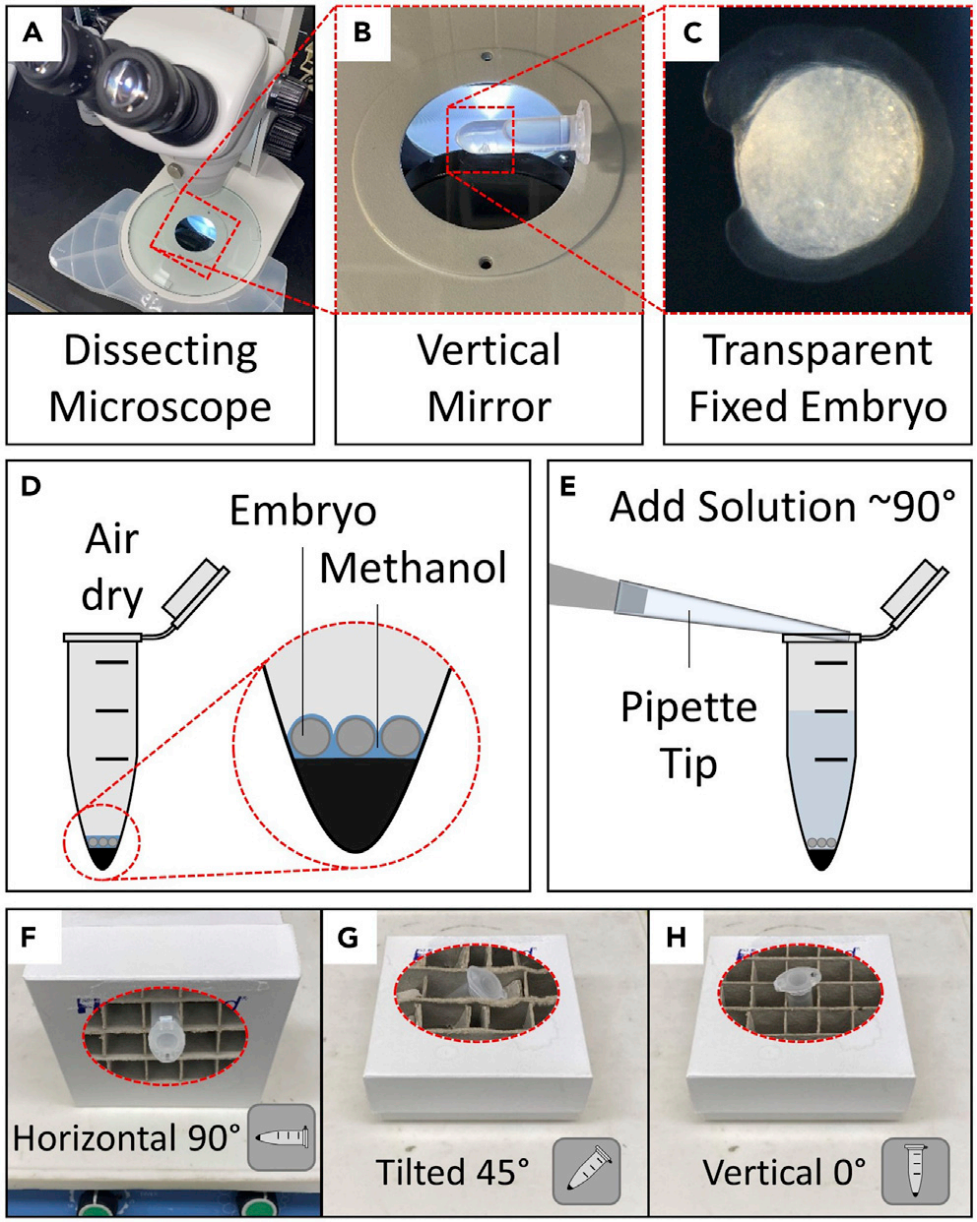
Critical experimental steps (A and B) (A) Image of dissecting microscope and (B) its mirror position. (C) A representative image of fixed embryo with transparent tissue. (D) Schematic representation how tubes should be seen after air dry. (E) Pipette tip position (horizontal) while adding a solution to prevent damage of embryos. (F–H) (F) Horizontal, (G) tilted, and (H) vertical positions of tube in a box during shaking on the shaker or incubation in the oven.

**Figure 2:**
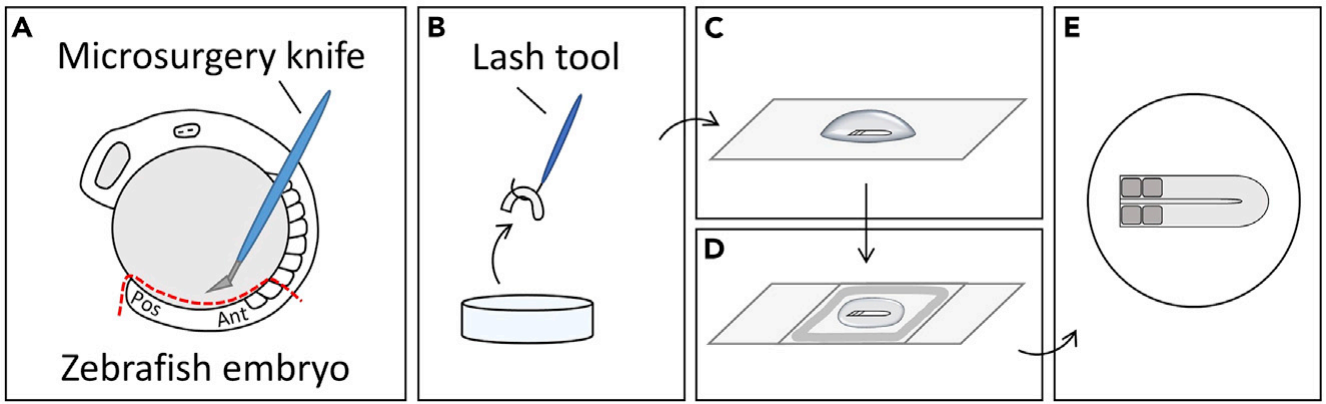
Flat-mounted sample preparation (A) Cut PSM from embryo using microsurgery knife. (B) Transfer tissue from petri dish into a drop of ProLong Gold antifade reagent on a glass slide. (C) Flattened tissue in the reagent. (D) Apply nail polish and fix the sample with a coverslip. (E) Dorsoventral view of a flat-mounted PSM tissue with couple of somites.

**Figure 3:**
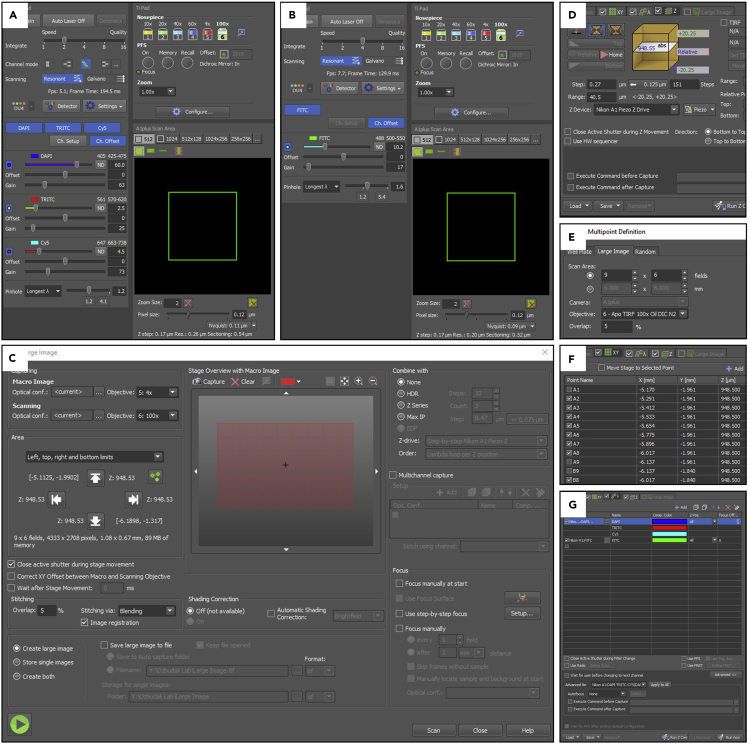
Microscopy settings for imaging (A and B) Microscopy settings for (A) DAPI-TRITC-CY5 and (B) FITC channels. Use separate optical configurations for DAPI-TRITC-CY5 and FITC because of different integrate levels. (C) “Scan Large Image” window, where left-top-right-bottom limits of the tissue and number of tiles in XY dimensions are detected. (D) Settings for Z layer in the relative symmetric mode. Z-step size should be 0.27 μm. (E) Window shows adding custom XY multipoints. (F) Positions of XY multipoints that covers the tissue. Remove unnecessary multipoints in case corner tiles are free of tissue. (G) Multichannel panel, which includes DAPI-TRITC-CY5 and FITC optical configurations.

**Figure 4:**
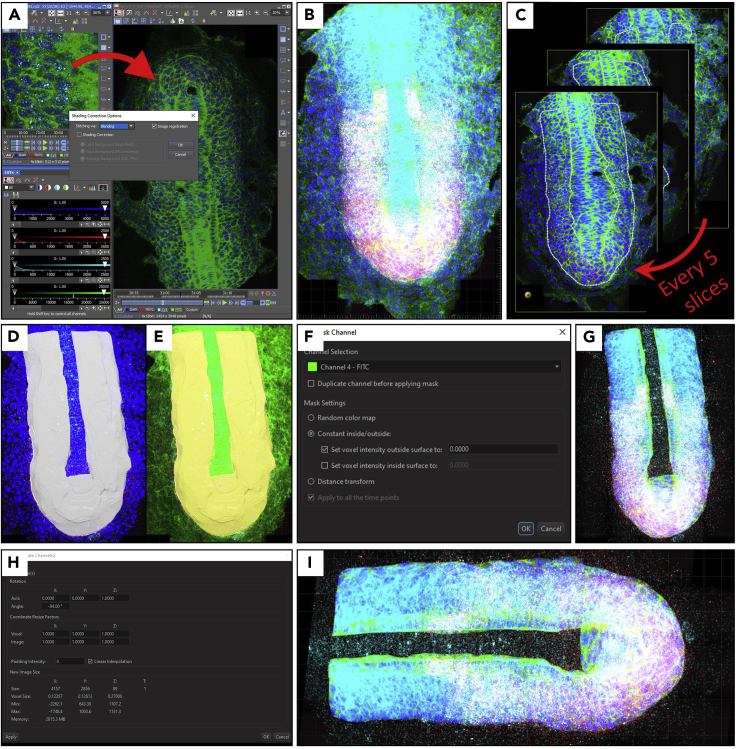
Surface creation and masking image (A) Stitch ND2 raw image. One of the *her1*^*ci301*^;*her7*^*hu2526*^ mutant embryos is used as a repesentative sample. (B) Converted stitched file to Imaris format. (C) Select tissue format in every 5 slices using manual surface creation. (D and E) Created surface to mask DAPI (nucleus) and FITC (membrane) channels. (F) Use “Mask All” in the “Tab Edit”. Set voxel intensity outside surface to zero “0”. (G) Masked tissue. (H and I) (H) Use “free rotate” window to make image anterior-posterior view (I) from left to right.

**Figure 5:**
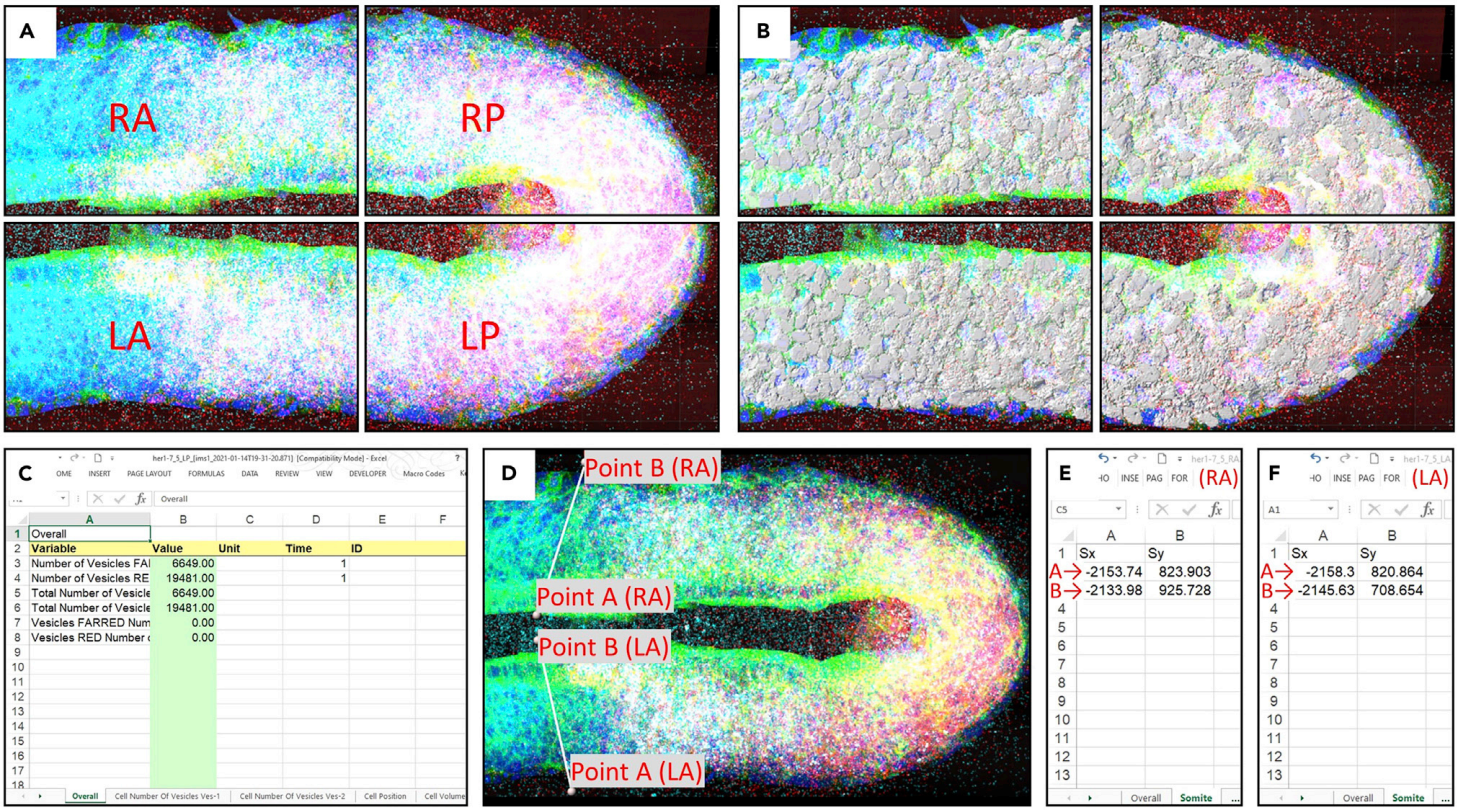
Cell segmentation for analysis and finding PSM-somite border (A) Split image into four images using “Crop 3D”. One of the *her1*^*ci301*^;*her7*^*hu2526*^ mutant embryos is used as a repesentative sample. (B) Segmented cells are seen in the Right-Anterior, Right-Posterior, Left-Anterior, and Left-Posterior of the tissue. (C) An example of XLS file, which includes statistics of “Cell Number of Vesicles”, “Cell Position”, and “Cell Volume”. (D) Measurement points for anterior PSM border. (E and F) Add X and Y data of the points in the “Somite” sheet of the (E) left-anterior and (F) right-anterior XLS file.

**Figure 6:**
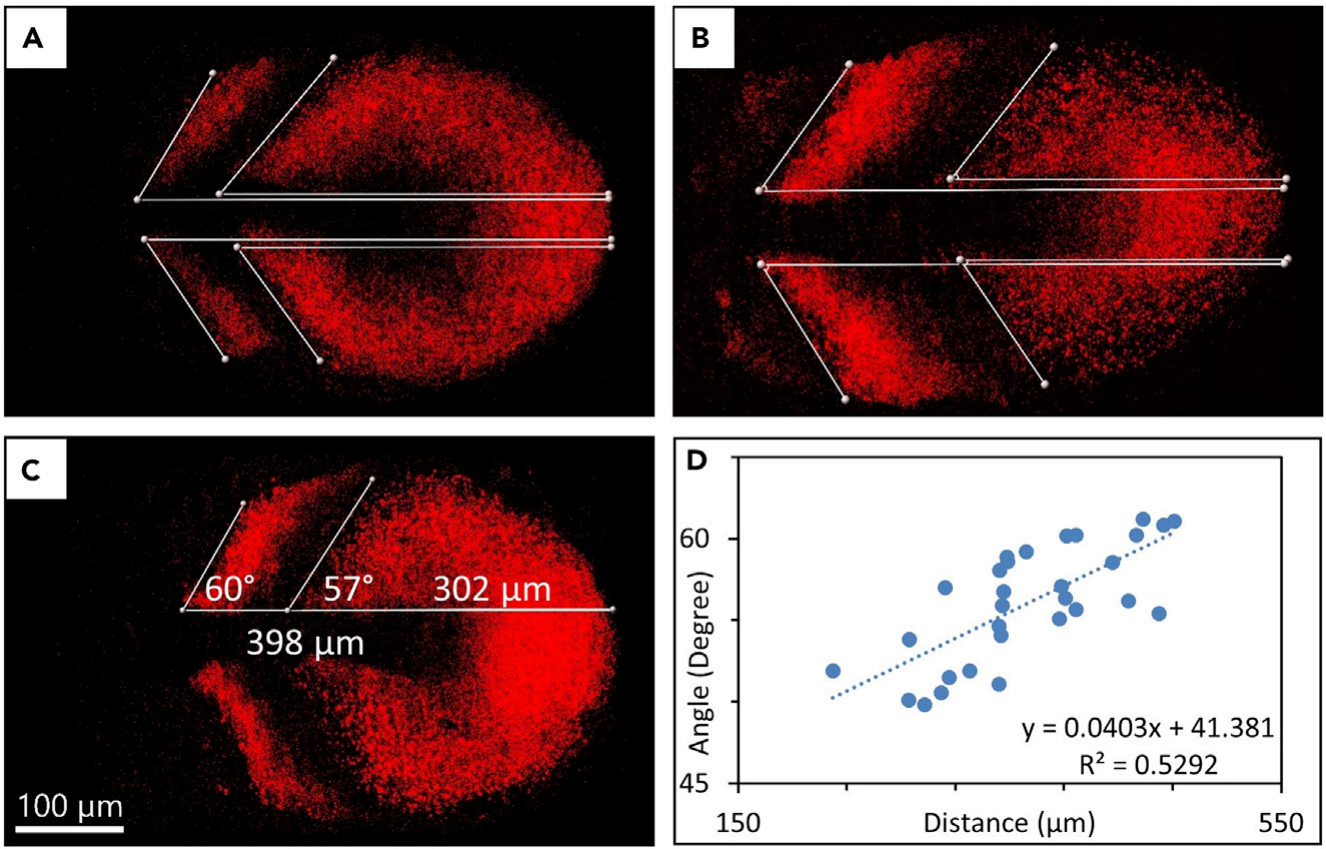
Measuring angle of expression stripes along the posterior-anterior axis (A–C) Measuring angle of expression stripes along the posterior-anterior axis and linear fitted equation for angle data (A), (B), and (C) show different wild-type samples as an example for measuring angle and distance of stripe to tailbud. Scale bar is 100 µm. (D) Plot “Angle vs Distance (μm)” graph to fit an equation, where the slope and Y-intersected data provide “*delta_angle*” and “*angle*”, respectively.

**Figure 7:**
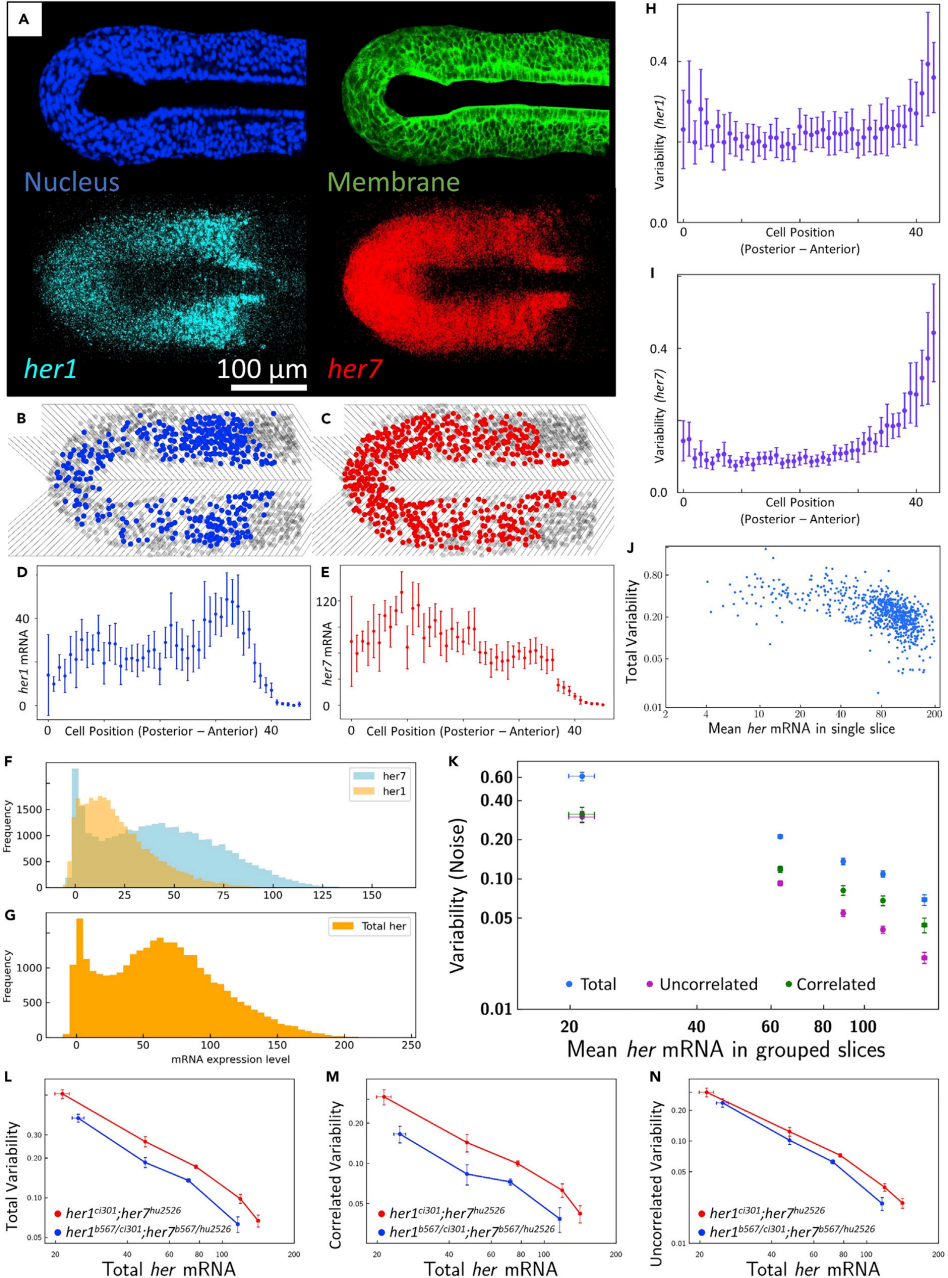
Example outcomes of images, RNA and variability (noise) distributions (A–N) (A) Masked nucleus (blue) and membrane (green) frames, and 3D projection of *her1* (cyan) and *her7* (red) transcripts of a *her1^ci301^*;*her7^hu2526^* mutant embryo. Scale bar is 100 µm. (B) and (C) show heatmaps of cells containing *her1* (blue) and *her7* (red) that are higher than an arbitrary threshold in single-cell-wide slices, respectively. (D) and (E) show *her1* (blue) and *her7* (red) RNA distribution along the posterior-anterior axis of the right-side of the embryo. Error bars are two standard errors of mean (SEM). (F) and (G) show the frequency histograms of *her1* (transparent orange) and *her7* (transparent blue), and total her (opaque orange) RNA expression per cell of *her1^ci301^*;*her7^hu2526^* mutant embryos (n=17, N=2), respectively. N is the number of independent experiments; n is the number of embryos. (H) and (I) show the average transcription variabilities of *her1* and *her7* per slice along the posterior-anterior axis. Error bars are two SEM. (J) shows total variability of mean *her* RNA levels of each single slice. (K) shows average of total (blue), correlated (green), and uncorrelated (purple) variabilities versus binned mean *her* RNA levels of slices. Error bars are two SEM. (L), (M), and (N) show total, correlated, and uncorrelated variability plots of multiple genetic backgrounds, *her1^ci301^*;*her7^hu2526^* (red, n=17, N=2) and *her1^b567/ci301^*;*her7^b567/hu2526^* (blue, n=12, N=2) mutant embryos. Adapted from Zinani et al.^3^ N is the number of independent experiments; n is the number of embryos. Error bars are two SEM.

**Figure 8:**
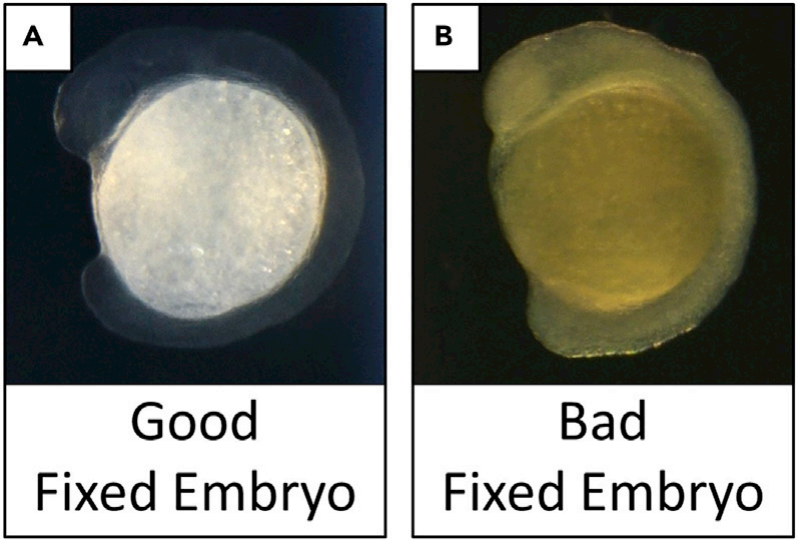
Comparison between “good” and “bad” fixed embryos

***Note:*** Store the rest of 4% PFA at 4°C as it is going to be used in Day 3 and Day 6.

***Note:*** Before the end of fixation (after ∼45 min), check transparency of embryos under dissecting microscope with dim light and from the black side of the mirror (see Figures 1A and 1B). Continue experiment only if the embryos are transparent (see Figure 1C). If the embryos are not transparent, it means the PFA is not good (see Figure 8B).

5. Remove PFA. Rinse with 1 mL PBST-0.1%.
6. Add 2 mL PBST-0.1%. Shake slowly for 5 min.

***Note:*** In the meantime, mix 3 mL PBST-0.1% and 3 mL Methanol into a 60 mm petri dish.

7. Dechorionate embryos in the petri dish (3 mL PBST-0.1% + 3 mL Methanol) for 5 min in a dark room with the weakest microscope light.
8. Transfer embryos back to the tube. Rinse them with 1 mL Methanol.
9. Add 2 mL Methanol. Shake slowly for 5 min.
10. Refresh medium with 2 mL Methanol. Store tube for 24 h at −20°C.

### Day 2: Probe hybridization

**Timing: 4 h**

This section describes the steps for smFISH probe hybridization developed from the RNAscope technology and Gross-Thebing et al.^5^

***Note:*** Start this step afternoon (∼1 pm). Set the oven to 40°C. Take tube out, wait until it reaches to 20°C–25°C.

11. Transfer embryos to 1.5 mL flat bottom microcentrifuge tube with glass pipette.

***Note:*** Using 1.5 mL flat bottom microcentrifuge tube is important for evenly staining of 15 embryos.

***Note:*** Using glass pipette is important as embryos might get stuck to the side of plastic tips.

12. Remove methanol as much as you can. Do not touch / damage embryos.

***Note:*** To remove solution as much as you can, first remove solution with 1000-μL filtered tip until less than 250 μL of the solution remains. Then hold the tube tilted, and remove the rest of the solution along the side of the tube taking care to avoid touching the embryos.

13. Air dry: leave tube open for methanol evaporation until embryos don’t contact each other with methanol (∼5–20 min) (see Figure 1D).

**CRITICAL:** Don’t let the embryos dry. If embryos’ color turns into yellow-orange color, which means they are dried, it becomes harder to flat mount embryos later.

14. Add 1 drop of RNAscope Protease-III pre-treatment solution. Gently tap the tube to sink embryos down. Wait for 20 min at 20°C–25°C.

**CRITICAL:** Protease makes embryos so fragile. After this step, be more careful and gentle with handling embryos till the end of experiment. From now on, never touch embryos with a tip while removing solutions. Add new solutions slowly from the inner side of the tube with ∼90° pipette tip (see Figure 1E). Always use either filtered or autoclaved tips. Shakings should be done at the slowest speed (i.e., approximately 20 rpm).

***Note:*** Prepare PBST-0.01% and fresh prehybridization buffer.

***Optional:*** You can prepare PBST-0.01% and fresh prehybridization buffer before starting the 11^th^ step on Day 2.

15. Rinse with 1 mL PBST-0.01% gently.
16. Add 1 mL PBST-0.01%. Shake tube slowly in a horizontal position for 5 min (see Figure 1F).
17. Repeat the previous step for 2 more times.
18. Remove all solution, add fresh 250 μL prehybridization buffer. Place tube in a box with 45° tilted position (see Figure 1G) and incubate it in oven at 40°C for 2 h.

***Note:*** Before 20 min end of prehybridization, take probes out from 4°C, warm them in oven (40°C) for 10 min, then cool to 20°C–25°C. Prepare probe mixture in a 1.5 mL microcentrifuge tube: 1 μL *Dr-her1-LE2*-C3 into 50 μL *Dr-her7*.

***Note:*** While C1 probes are ready to use, C2 and C3 probes are shipped as a 50× concentrated stock. Thus, drop couple of droplets from C1 probe into a sterile microcentrifuge tube and use it as C1 aliquot.

**CRITICAL:** Before preparing the probe mixture, make sure all probes are completely dissolved.

19. Remove all solution. Add 15 μL probe mixture. Gently tap the tube. Place tube in a box vertically (see Figure 1H) and incubate it in oven at 40°C for 16 h.

**CRITICAL:** Make sure all embryos are in a row at the bottom of the tube for an even staining.

***Note:*** Start probe hybridization at ∼6 pm and end it next day by ∼9 am to ensure sufficient hybridization and maintain consistency between each experiment.

***Note:*** Add 1 μL probe mixture per embryo. If you have less than 10 embryo in a tube, add 10 μL probe mixture at a minimum volume. Do not include more than 15 embryos in a single tube.

### Day 3: Amplifications and washes

**Timing: 10 h**

This experimental day includes four signal amplification steps in between a series of washings. To detect target RNAs, probes are hybridized to amplification molecules, which make target RNAs to be visible in a fluorescent channel via binding of dye-labeled probes.

***Note:*** Before starting this step, prepare 50 mL SSCT (0.2× SSC, 0.01% Tween 20). Place 4% PFA at 20°C–25°C. Be careful and gentle to embryos since they are fragile. Solutions should be added slowly from the inner side of the tube with ∼90° pipette tip (see Figure 1E). All the shakings should be done at the slowest speed (∼20 rpm) at 20°C–25°C. Shake tube in a horizontal position (90°, see Figure 1F) if it contains ∼1 mL solution, in a tilted position (45°, see Figure 1G) if it contains 200–400 μL, or in a vertical position (0°, see Figure 1H) if it contains less than 200 μL.

20. Rinse with 1 mL SSCT.
21. Add 1 mL SSCT. Shake tube slowly in a horizontal position for 15 min.
22. Repeat the previous step for 4 more times.
23. Remove all SSCT.
24. Rinse with 1 mL 4% PFA.
25. Add 1 mL 4% PFA. Shake tube slowly in a horizontal position for 10 min.

***Note:*** Store 4% PFA at 4°C.

26. Remove 4% PFA as much as you can. Rinse with 1 mL SSCT.
27. Add 1 mL SSCT. Shake tube slowly in a horizontal position for 15 min.

***Note:*** Take Amp 1 out to 20°C–25°C for 30 min before applying it.

28. Repeat the previous step for 2 more times.
29. Remove all SSCT.

**CRITICAL:** Remove all solution before amplification reagents to ensure staining quality and comparability.

30. Add one drop of Amp 1 (∼20 μL). Tap gently to sink embryos down. Place tube vertically and incubate it in an oven at 40°C for 30 min.
31. Rinse with 1 mL SSCT.
32. Add 1 mL SSCT. Shake tube slowly in a horizontal position for 15 min.
33. Repeat the previous step for 4 more times.

***Note:*** Take Amp 2 out to 20°C–25°C for 30 min before applying it.

34. Remove all SSCT.
35. Add one drop of Amp 2 (∼20 μL). Tap gently to sink embryos down. Place tube vertically and incubate it in an oven at 40°C for 15 min.
36. Rinse with 1 mL SSCT.
37. Add 1 mL SSCT. Shake tube slowly in a horizontal position for 15 min.

***Note:*** Take Amp 3 out to 20°C–25°C for 30 min before applying it.

38. Repeat the previous step for 2 more times.
39. Remove all SSCT.
40. Add one drop of Amp 3 (∼20 μL). Tap gently to sink embryos down. Place tube vertically and incubate it in an oven at 40°C for 30 min.
41. Rinse with 1 mL SSCT.
42. Add 1 mL SSCT. Shake tube slowly in a horizontal position for 15 min.

**Table 2:** Color module options for Amp 4 Alt fluorescent labels

Probe channel ID	Amp 4 Alt A-FL	Amp 4 Alt B-FL	Amp 4 Alt C-FL
C1	Alexa 488 (Green)	Atto 550 (Red)	Atto 550 (Red)
C2	Atto 550 (Red)	Alexa 488 (Green)	Atto 647 (Far Red)
C3	Atto 647 (Far Red)	Atto 647 (Far Red)	Alexa 488 (Green)

**Table 3:** Microscopy settings

Parameter	Setting
Objective	100× Oil DIC
Numerical Aperture	1.49
Refractive Index	1.515
Zoom factor	2
Z-step size	0.27 μm
Step	80–150
Pinhole size	76.63 μm
Sectioning	0.52 μm
Image format	512 × 512 px
XY pixel size	0.12 μm
Scanning	Resonant
Direction	Bi-Direction

**Table 4:** Channel settings

Channel ID	Target	Excitation (nm)	Emission (filter set)	Laser power	PMT HV	PMT offset	Integrate
Channel 1 (DAPI)	Nucleus	405	450/50	N/A	N/A	0	2
Channel 2 (TRITC)	*her7*	561	595/50	2.5	25	0	2
Channel 3 (Cy5)	*her1*	647	700/75	4.5	73	0	2
Channel 4 (FITC)	Membrane	488	525/50	N/A	N/A	0	4

**Table 5:** Cell creation parameters

Parameter	Setting
Detection Type	Nucleus, Cell and Vesicles
Nucleus Diameter	3 μm
Smoothing (Nucleus)	Enabled
Filter Width (Nucleus)	0.3 μm
Background Subtraction (Nucleus)	Enable
Sphere Diameter (Nucleus)	1.2 μm
Split Nuclei by Seed Points	Enabled
Filter Nuclei Seed Points:	Disabled
Nuclei Threshold (Background Subtraction)	Automatic
Filter Nuclei: Nucleus Number of Voxels	8543.12 and 6.77591e^4^
Cell Detection Type	Cell Boundary from Cell Membrane
Cell Smallest Diameter	0.25 μm
Membrane Detail (Cell)	0
Filter Cells: Cell Volume	150 μm^3^ and 450 μm^3^
Estimated Diameter (Vesicle)	0.5 μm
Background Subtraction (Vesicle)	Enabled
Different Vesicle Size	Disabled
Filter Vesicles: Quality	Variable for each probe

**Table 6:** Required cell statistics

Statistical parameter
Cell Number of Vesicles
Cell Position (X,Y,Z)
Cell Volume

***Note:*** Take Amp 4 Alt B out to 20°C–25°C for 30 min before applying it.

***Alternatives:*** Choose one of the alternate fluorescent color modules (Amp 4 Alt A, B, or C) that you want to label probes with different fluorophores (see Table 2). In this experiment, since we stain nucleus with Hoechst and membrane with GFP, we choose Amp 4 Alt B to label C1 (*her7*) and C3 (*her1*) probes with red and far red, respectively (see Table 1).

43. Repeat the previous step for 2 more times.
44. Remove all SSCT.
45. Add one drop of Amp 4 Alt B (∼20 μL). Tap gently to sink embryos down. Place tube vertically and incubate it in an oven at 40°C for 15 min.

**CRITICAL:** Since Amp 4 contains fluorescent dye, keep tube covered with aluminum foil at all times to ensure staining quality and comparability.

46. Rinse with 1 mL SSCT.
47. Add 1 mL SSCT. Shake tube slowly in a horizontal position for 15 min.
48. Repeat the previous step for 2 more times.
49. Remove all SSCT.
50. Rinse with 1 mL 4% PFA, then add 1 mL 4% PFA.
51. Store embryos, 4% PFA and SSCT (if any) at 4°C for 16 h.

***Note:*** In order to avoid air bubbles, start preparing PBSTX one day before immunohistochemistry staining. Mix 5 mL 10× PBS with ∼30 mL DEPC water, then add 500 μL Triton X-100. Mix well and leave it at 20°C–25°C. Top it up to 50 mL with DEPC water on the next day.

### Day 4: Primary antibody staining

**Timing: 4 h**

The following steps describe primary antibody staining for IHC on the fourth day of experiment.

***Note:*** Start this step afternoon (∼1–2 pm). Top up PBSTX to 50 mL that was prepared the day before. Final concentration would be 1× PBS 1% Triton X-100.

**CRITICAL:** Do not start too early or late to make sure enough incubation time and to finish the last step at around 6 pm as embryos are very fragile and buffers are pretty harsh.

52. Remove 4% PFA. Rinse with 1 mL PBSTX.
53. Add 1 mL PBSTX. Shake tube slowly in a horizontal position for 5 min.

***Note:*** Prepare permeabilization buffer (995 μL PBSTX + 5 μL Triton X-100 for each tube). Don't introduce bubbles.

54. Repeat the previous step for 2 more times.
55. Remove PBSTX. Add 1 mL permeabilization buffer. Shake tube slowly in a horizontal position for 1 h at 20°C–25°C.

***Note:*** Be careful, don't add air bubbles.

***Note:*** Prepare blocking buffer PBSTXBG: 0.2 g BSA + 10 mL PBSTX + 100 μL GS.

56. Remove permeabilization buffer. Add 1 mL PBSTXBG. Shake tube slowly in a horizontal position for 2 h at 20°C–25°C.

***Note:*** Prepare primary antibody solution: 180 μL PBSTXBG + 1 μL Chicken anti-GFP. Store the rest of PBSTXBG at 4°C.

57. Remove PBSTXBG as much as you can (probably 20 μL left). Add 180 μL primary antibody solution. Final concentration would be 1:200 (10 μg/mL). Shake tube slowly with 45° tilted position for 20–24 h at 4°C in a cold room.

### Day 5: Secondary antibody staining

**Timing: 1 h**

The following steps describe secondary antibody staining for IHC on the fifth day of experiment.

***Note:*** Start this step afternoon (∼4 pm).

58. Remove primary antibody solution. Rinse with 1 mL PBSTX. Add 1 mL PBSTX. Shake tube slowly in a horizontal position for 10 min at 20°C–25°C.
59. Repeat the previous step for 2 more times.
60. Remove PBSTX. Add 1 mL PBSTXBG. Shake tube slowly in a horizontal position for 10 min at 20°C–25°C.

***Note:*** Prepare secondary antibody solution: 380 μL PBSTXBG + 2 μL Alexa Fluor 488 anti-Chicken + 1.1 μL Hoechst 33342 per tube.

61. Remove PBSTXBG as much as you can (probably 20 μL left). Add 380 μL secondary antibody solution. Final concentrations would be 1:200 (10 μg/mL) for secondary antibody, and 27.5 μg/mL for Hoechst. Shake tube slowly with 45° tilted position for 16 h at 4°C in a cold room.

### Day 6: Sample preparation

**Timing: 1 h**

This section describes steps how to prepare flat-mounted samples for confocal imaging.

***Note:*** Take 4% PFA and SSCT out and warm up to 20°C–25°C.

62. Remove secondary antibody solution. Rinse and wash embryos once with SSCT. Shake tube slowly in a horizontal position for 7 min at 4°C in a cold room.
63. Rinse and add 1 mL 4% PFA.

**Pause point:** Embryos can be stored in dark at 4°C for up to 7 days.

64. Place tube on ice. Use SSCT for sample preparation in a 60-mm petri dish.

**CRITICAL:** Avoid light as much as possible during sample preparation. Work in a dark room with the weakest microscope light. Turn microscope mirror to ∼90 degree against desk for a minimum light exposure.

65. Place one embryo into petri dish using a transfer pipette. First, cut from the head with a microsurgical knife. Then do little cuts on the skin to remove yolk from the tissue (see Figure 2A). Use a lash tool to gently remove excess yolk. Cut until the presomitic mesoderm (PSM) and 1–2 somites are left.

**CRITICAL:** Remove all yolk to ensure integrity of the PSM because yolk proteins interfere with fluorescent proteins.

66. Place one drop of ProLong Gold antifade reagent on a glass slide and transfer tissue into it using a lash tool (see Figure 2B).
67. Make sure the tissue is dorsoventrally flattened (see Figure 2C).
68. Apply transparent nail polish and then fix with the coverslip on top gently (see Figures 2D and 2E). Wait for 5 min for nail polish to dry before starting the imaging.

**CRITICAL:** Apply consistent amount of nail polish to control the PSM tissue thickness and imaging quality.

**CRITICAL:** Keep prepared slides in dark at 4°C, and complete imaging on the day of sample preparation.

### Imaging

**Timing: 12 h to 7 days**

This section describes steps and parameters for confocal imaging of flat-mounted smFISH and IHC stained samples (see Tables 3 and 4 for microscopy and channel settings).

***Note:*** Resonant scanner confocal microscope is recommended to perform smFISH imaging. In our experiment, we used Nikon A1R HD confocal on TiE microscope with an Apo TIRF 100× Oil DIC N2 objective. For an equal comparison, do not change laser settings in the channels for RNA probes among the samples. On the other hand, you can set different laser powers and voltage in membrane and nucleus channels in order to achieve better cell segmentation. For an even imaging quality, periodically check laser strength.

***Note:*** Find appropriate laser settings for nucleus and membrane channels of each sample at the most anterior part of the tissue as the region of interest (i.e., PSM tissue) might be affected due to photobleaching. Signals should not be oversaturated but high enough to be detected in Imaris cell segmentation. In our case, 50–60 laser power and ∼60 PMT HV in DAPI channel, and 2–10 laser power and 10–20 in GFP channel depends on membrane injection and staining quality.

**CRITICAL:** As GFP can bleach quickly, avoid high laser power.

***Note:*** Since Channel 4 has a different integrate level, save it as a separate optical configuration in Nikon’s confocal NIS-Elements software.

69. Put oil onto the lens of 100× objective.
70. Place specimen onto the stage.
71. Find focus plane by ocular lenses.
72. Check nucleus (Hoechst) and membrane (GFP) signal at the most anterior part of the tissue and find appropriate laser power and PMT HV (see Figures 3A and 3B).
73. Open Acquire>Scan Large Image. Select “Left, top, right and bottom limits” in the Area panel.
74. Set stitching overlap as 5% (see Figure 3C).
75. In the DAPI channel (for nucleus), run the Live camera, locate sample’s XY limits. Then, in the same channel, set the Z range at the positions where there is no signal by using Top and Bottom buttons in the Z panel in the ND Acquisition window. Thus all the tissue is covered in the image dorsoventrally. Press Home (middle Z position), go to symmetric mode and press Relative button (see Figure 3D). Then go to middle XY position and close “Scan Large Image” window.

***Note:*** Keep in mind the number of X and Y fields to use it while adding custom XY multipoints in the following step.

76. Go to XY panel in the ND Acquisition window. Select “include Z”. Press Custom button. Under the Large Image section, select 100× objective, write number of X and Y fields, and 5% as overlap area (see Figures 3E and 3F).

***Note:*** This step adds custom XY multipoints, which cover the tissue, at the middle Z layer.

***Note:*** Deselect any multipoint if it doesn’t include the tissue because of rotated sample mounting.

77. Add optical configurations in to the λ (multichannel) panel in the ND Acquisition window (see Figure 3G).

***Note:*** While one of the optical configurations includes DAPI-TRITC-Cy5 channels, the other one includes FITC channel because they have different signal integration parameters.

78. Run image.

### Analysis

**Timing: 2 to 7 days**

This section describes steps to analyze images. First, prepare images for analysis (i.e., creating surface, masking and cell segmentation). Then, using cell positions and single transcript levels per cell, run “variability (noise)” codes for the RNA and variability distribution.

#### Masking and cell segmentation

79. Open multipoint ND2 file in NIS Elements. Go to “Image > ND Processing > Stitch Multipoint to Large Image”. Stitch mutipoints via blending without shading correction. Save the stitched file (see Figure 4A).
80. Use “Imaris File Converter” to convert stitched ND2 file to IMS file (see Figure 4B).
81. Open IMS file in Imaris software. Use “Surface” wizard to mask nucleus and membrane of the tissue.
82. In the 3D view, select “Surfaces”. Skip automatic surface creation and click edit manually.
83. Choose “Manual” resolution with highest X-Y size.
84. Turn on “slicer rendering for selected object”.

***Alternatives:*** Deselect “Volume” view in the Surpass Tree list.

85. Click “Draw” and select the region of interest in every 5 slices from top to bottom of the image (see Figure 4C).

***Note:*** If the tissue borders change dramatically along the z-axis (e.g., dorsal side of the tissue, where tissue narrows immediately), select the tissue borders in every 2–3 slices instead of 5.

**CRITICAL:** Select precisely from the border of the PSM cells. Make sure to include adaxial cells in the PSM and S1 somite, but exclude notochord, neural tube, and skin cells.

***Alternatives:*** Select out of PSM and mask it out later.

86. Click “Create Surface” (see Figures 4D and 4E). Then click “Mask All” in the “Tab Edit”. Choose Nucleus channel and set voxel intensity outside surface to zero. Repeat it for Membrane channel too (see Figures 4F and 4G).

***Note:*** Deselect “Duplicate channel before applying mask”.

***Alternatives:*** If you selected outside of the tissue, set voxel intensity inside surface to zero.

87. Rotate the image from “Image Processing > Free Rotate” to make it horizontal view from anterior to posterior by using appropriate angle. Set X and Y axis as “0” and Z axis as “1” in the rotation axis (see Figures 4H and 4I).
88. Split the image into four images from “Edit > Crop 3D”, and rename files of the left-anterior, left-posterior, right anterior, and right-posterior parts as ending “_LA”, “_LP”, “_RA”, and “_RP”, respectively (see Figure 5A).
89. For cell segmentation, click “add new cells” (see Table 5 for cell creation parameters).
90. Select detection type as “Detect Nucleus, Cell and Vesicles”.
91. Select nucleus channel. Set nucleus diameter as 3 μm. Apply “smoothing” (filter width 0.3 μm), “background subtraction” (sphere diameter 1.2 μm) and “split nuclei by seed points”.
92. Set automatic nuclei threshold (background subtraction), Then apply filter nuclei, “Nucleus Number of Voxels” between 8543 and 6.78e^4^.
93. Select cell detection type as “Detect Cell Boundary from Cell Membrane”. Set “cell smallest diameter” as 0.25 μm and “membrane detail” as zero.
94. Apply “Cell Volume” filter between 150 μm^3^ and 450 μm^3^.
95. As for vesicles, select channels for each RNA, set estimated dimeter as 0.5 μm, and apply background subtraction.
96. For each vesicle type, add “quality” filter, which is supposed to be high enough to detect one vesicle for each single RNA punctum.

***Note:*** Cell creation parameters can be stored and used for a batch analysis in Arena later.

**CRITICAL:** Embryos within the same batch should have similar quality score.

97. After the end of cell segmentation (see Figure 5B), export all cell statistics to file in XLS format (see Figure 5C) from every split images (see Table 6 for required cell statistics).
98. Add a “Measurement Point” in the Surpass Tree list of the left-anterior and right-anterior files. Find anterior borders of the PSM tissue in each sample (see Figure 5D). Create “Somite” sheet in all anterior files (i.e., “*TITLE*_LA.xls” and “*TITLE*_RA.xls”). Add X-Y positions of two points in Cell A2-B2 for “Point A” and Cell A3-B3 for “Point B” in the “Somite” sheet (see Figures 5E and 5F).
99. Measure angle and distance from the tail end of expression stripes of the segmentation clock in the PSM in all embryos in each genetic background (see Figures 6A–6C). Then fit an equation to the data, i.e., “Angle_vs_Distance = *delta_angle* ∗ Distance + *angle*”, which provides “*delta_angle*” and “*angle*” to be used in “*GENOTYPE*_wVol.py” or “*GENOTYPE*_woVol.py” later for analysis (see Figure 6D).

**CRITICAL:** As the angles of stripes vary smoothly along the posterior-anterior axis, it is important to use comparable angles of slices with that of expression stripes.

#### Noise calculation

100. Transfer all XLS files into “XLS_to_XLSX” folder and convert XLS files to XLSX format using “xlsToxlsxIMARIS.py”.
101. Transfer anterior XLSX files (i.e., “*TITLE*_LA.xlsx” and “*TITLE*_RA.xlsx”) into “Anterior” folder and posterior XLSX files (i.e., “*TITLE*_LP.xlsx” and “*TITLE*_RP.xlsx”) into “Posterior” folder.
102. Run “separatePSM_volume_Green_farred.m” and “combineLRP_volume.m” in “Anterior” and “Posterior” folder, respectively, to calculate background from anterior files and combine sheets using MATLAB.

***Note:*** Make sure titles of vesicle sheets in the MATLAB codes are the same with the sheets in the XLSX files.

103. Transfer all “*TITLE*_LA_PSM.xlsx”, “*TITLE*_RA_PSM.xlsx”, “*TITLE*_LP_PSM.xlsx”, and “*TITLE*_RP_PSM.xlsx” files into a “MergedPSM” folder and merge files using “merge_excel_FileInDirectory_wo_Vol.py” for without volume correction and “merge_excel_FileInDirectory_w_Vol.py” for with volume corrected RNA levels.

***Note:*** Check background values at “bgSomiteCount.xlsx”. Each tissue has two rows of background values either coming from left or right side of anterior PSM. Additionally, while columns B-E show raw values for mean or variance of farred and red spots in the somitic tissue, columns F-I show volume corrected background values. For raw RNA per cell distribution analysis, copy and paste row values, either from left or right anterior files, between columns B-E for every tissue into “SampleInfo.xlsx” file in the “Input” folder of “without volume corrected” excel files. For volume-corrected RNA variability (noise) analysis, copy and paste values between columns F-I, either from left or right anterior files, for every tissue into “SampleInfo.xlsx” file in the “Input” folder of “volume corrected” excel files.

104. Rename “*TITLE*_merged_w_Vol_PSM.xlsx” or “*TITLE*_merged_wo_Vol_PSM.xlsx” files with an ascending order number ending like “*TITLE*_wv_*#.*xlsx” or “*TITLE*_wov_*#.*xlsx”. Transfer them into the same “Input” folder together with “SampleInfo.xlsx” file, which has background values for each tissue.

***Note:*** Edit “Python name” in Column B in the “SampleInfo.xlsx” file in accordance with “*TITLE*_wv_*#*.xlsx” or “*TITLE*_wov_*#*.xlsx” excel filenames like “*TITLE*_wv_*#*” or “*TITLE*_wov_*#*”.

105. Run “*GENOTYPE*_wVol.py” and “*GENOTYPE*_woVol.py” for each genetic background.

***Note:*** Make sure folder paths and filename formats are corrected in the python code.

***Note:*** If there is no stripy expression, use wild-type’s angle of expression and angle variation slope, which are 41.381 and 0.0403, respectively.

106. To compare transcriptional variabilities of different genetic backgrounds, run “COMPARE_GENETICBACKGROUNDS.py”.

***Note:*** Format: python COMPARE_GENETICBACKGROUNDS.py < number of genetic backgrounds> <number of bins for noise plots> <comparison outcome folder> <folders containing files to be compared (i.e., directory of “Output” folders of each genotype)> <genetic background names for labeling> <genetic background colors for plotting>

***Note:*** Example: python COMPARE_GENETICBACKGROUNDS.py 2 5 ../Compare/b567xher17_vs_her17/ ../wVol/b567xher17/Output/ ../wVol/her17/Output/ b567xher17 her17 b r.

## Expected outcomes

Example outcomes of images, RNA and variability (noise) distribution are shown in Figure 7. Masked nucleus and membrane frames, and 3D projection of *her1* and *her7* transcripts of *her1*^*ci301*^;*her7*^*hu2526*^ mutants are seen in Figure 7A. After splitting images into four images and running cell segmentation, run “*GENOTYPE*_woVol.py” code for background subtracted RNA distribution or “*GENOTYPE*_wVol.py” code for variability distribution results. Figures 7B and 7C show heatmaps for *her1* and *her7* containing cells in single-cell-wide slices, respectively. Cells, which have higher an arbitrary threshold, are plotted as blue or red circles. Figures 7D and 7E show RNA distribution along the posterior-anterior axis of the right hand side of the embryo. Figures 7F and 7G show the frequency histograms of *her1* and *her7*, and total *her* RNA expression per cell of 17 *her1*^*ci301*^;*her7*^*hu2526*^ mutant embryos. Using *her1* and *her7* RNA levels per cell, transcription variabilities based on cell positions or mean total RNA levels in single slices are plotted (see Figures 7H–7J). Figure 7K shows average of total, correlated (extrinsic), and uncorrelated (intrinsic) variability versus average of total *her* (*her1* + *her7*) RNA in grouped slices. Running “COMPARE_GENETICBACKGROUNDS.py” code gives variability plots of multiple genetic backgrounds, e.g., between *her1*^*ci301*^;*her7*^*hu2526*^ embryos (n = 17, N = 2) and *her1*^*b567/ci301*^;*her7*^*b567/hu2526*^ embryos (n = 12, N = 2) (see Figures 7L–7N, adapted from Zinani et al.^3^

## Limitations

The smFISH technique described in this protocol is limited to simultaneously detect up to two different RNA targets per cell. Two additional channels are spared to detect nucleus and membrane for proper cell segmentation in a compact tissue. Injection of large quantities of membrane-localized fluorescent protein RNA might cause abnormal morphology in few embryos. So one should not inject too much RNA.

## Troubleshooting

### Problem 1

Non-transparent embryo after fixation (Refer to day 1: fixation, step 4).

### Potential solution

Fixing embryos properly with PFA is a critical step prior to smFISH staining. If it has a pH different than 7.0, or if it is an old solution, it causes non-transparent tissue, which leads to high background staining signal. Prepare fresh PFA with a pH 7.0. Aliquot into 15 mL tubes and store at −20°C. Always avoid multiple freeze and thaw cycles.

### Problem 2

Yellow-orange colored embryos after air dry (Refer to day 2: probe hybridization, step 13).

### Potential solution

Do not let embryos dry. It is enough to wait until there is still some methanol left at the very bottom of the tube, but embryos should lose their contacts through methanol.

### Problem 3

Embryos might be broken (Refer to day 2: probe hybridization, step 14).

### Potential solution

After incubation of embryos with RNAscope Protease-III pre-treatment solution, embryos become more fragile. Be gentle after this step until the end of staining (day 6). Add solutions slowly (see Figure 1E). Use the lowest speed of rotary shaker. Do not touch embryos with pipette tip etc.

### Problem 4

No signal in RNA channels after smFISH staining (Refer to imaging, step 71).

### Potential solution

• Always use filtered tips and RNase free solutions. Otherwise smFISH probes or RNAs might be degraded.
• Check probe hybridization and amplification steps (Refer to day 2 and day 3). Make sure embryos were at the bottom of the tube and covered by the solutions in each step. Tap tube gently to sink embryos if they are floating after adding amplification solutions.

### Problem 5

Localized blockage of signal in the tissue (Refer to imaging, step 72).

### Potential solution

• If you image dorsoventrally, the yolk proteins might interfere with fluorescent proteins. Make sure you cleaned yolk from the tissue.
• Use soft lash tool. Do not damage the tissue while deyolking.

### Problem 6

No or low GFP signal (Refer to imaging, step 72).

### Potential solution

• Make sure you injected enough amount of membrane-localized GFP RNA (400 pg) (Refer to before you begin, step 7).
• Pick embryos with a high GFP signal before fixation. Do not continue if you don’t have GFP positive embryos (Refer to day 1: fixation, step 1).
• Do not excite the tissue too much to find appropriate laser power for FITC channel as GFP can bleach quickly (Refer to imaging, step 72).

## Resource availability

### Lead contact

Further information and requests for resources and reagents should be directed to the lead and corresponding author Ertuğrul M. Özbudak (Ertugrul.Ozbudak@cchmc.org).

### Materials availability

This study did not generate new unique reagents.

## Data Availability

Matlab and Python codes, mentioned in the Analysis section, are provided at GitHub (GitHub: https://github.com/ozbudak/STARMethods_2022) (Zenodo: https://doi.org/10.5281/zenodo.7449960). The data used in Figures 4, 5, 6 and 7 were previously published and deposited online, and are publicly available in the BioStudies database (the accession numbers are BioStudies: S-BSST434, S-BSST847).^2^^,^^3^
